# Identification of RAGE Inhibitors from *Curcuma caesia* Roxb: Implications in Treatment against Diabetic Complications

**DOI:** 10.2174/0115733998416568251122070701

**Published:** 2026-01-20

**Authors:** Priyakshi Nath, Anil K. Pasupulati, Rupshikha Nath, Rajat Nath, Mriganka Das, Deepa Nath, Jagajjit Sahu, Sibashish Kityania, Anupam Das Talukdar

**Affiliations:** 1 Department of Life Science and Bioinformatics, Assam University, Silchar, Cachar, Assam, 788011, India;; 2 Department of Biochemistry, University of Hyderabad, 500046, India;; 3 Department of Biotechnology and Microbiology, School of Natural Sciences, Techno India University Tripura (TIUT), Agartala, 799004, Tripura;; 4 Department of Botany, Gurucharan College, Silchar, Cachar, Assam, 788004, India;; 5 Bioinformatics Nexus Network (BNSquare), GyanArras Academy, Bhubaneshwar, Khurda, Odisha, 751003, India

**Keywords:** *Curcuma caesia.*, AGEs, RAGE, diabetic nephropathy, lappaol A, piperaduncin B

## Abstract

**Introduction:**

Diabetic nephropathy (DN) is a progressive renal complication that significantly contributes to end-stage renal disease. Hyperglycaemia contributes to the formation of advanced glycation end-products (AGEs). The interaction between AGEs and their receptor (RAGE) plays a key role in the progression of DN. RAGE activation increases oxidative stress and promotes inflammation, thereby evoking cellular and molecular damage. Together, these events result in kidney injury and varying degrees of proteinuria. This study aims to evaluate the drug-like properties of potential natural compounds derived from *Curcuma caesia* and their potential effectiveness against DN.

**Methods:**

This study investigates the antioxidant properties of *Curcuma caesia* (CC) rhizome extracts, alongside *in silico* methodologies including molecular docking, QSAR, and ADMET analysis to identify potential metabolites.

**Results:**

In this study, we examined the potential of phytochemicals identified from the rhizome extracts of *Curcuma caesia* (CC) that may mimic AGEs and inhibit RAGE activation. We assessed whether these phytochemicals could prevent ROS accumulation and inflammation, thereby providing renoprotection in a diabetic milieu. Using molecular docking and ADMET analysis, we identified two compounds, Lappaol A and Piperaduncin B, in the methanolic extract of CC, which demonstrated a stronger affinity for interacting with RAGE than the AGE compound MODIC and the RAGE inhibitor Azeliragon.

**Discussion:**

Since the interaction between AGEs and RAGE contributes to major pathological events in the development of DN, inhibiting this interaction could be a valuable therapeutic strategy against DN and other AGE-mediated pathologies such as retinopathy and neuropathy. Virtual screening of the identified compounds revealed that Lappaol A and Piperaduncin B effectively bind to RAGE and may interrupt RAGE activation, thereby potentially slowing the progression of DN.

**Conclusion:**

These natural compounds exhibited promising drug-like characteristics against the target protein RAGE and may serve as lead compounds for the development of RAGE inhibitors. The study recommends further *in vitro* and *in vivo* investigations to assess the therapeutic potential of these identified compounds in the treatment of diabetic nephropathy.

## 1 INTRODUCTION

Diabetic nephropathy (DN) is one of the major complications of diabetes and is the leading cause of chronic kidney disease and end-stage renal disease (ESRD) [1]. The global prevalence of DN can be attributed to the substantial rise in diabetes cases worldwide. In 2021, 529 million people were reported to be suffering from diabetes globally, and this number is projected to exceed 1.31 billion by 2050 [2]. About 30% of individuals with Type 1 diabetes and 40% of individuals with Type 2 diabetes develop DN [3]. Microalbuminuria in diabetic patients is a key indicator of the early onset of DN [4]. Microalbuminuria reflects damage to the glomerular filtration barrier (GFB), which consists of the inner glomerular endothelial cells, the middle glomerular basement membrane, and the outer layer of podocytes.

The EDIC (Epidemiology of Diabetes Interventions and Complications) study, an observational follow-up of the DCCT (Diabetes Control and Complications Trial), reported that poor blood sugar control is associated with a high incidence of diabetic complications, even when strict glycaemic control is attempted after diagnosis. Persistent hyperglycaemia can cause significant organ damage by stimulating the production of AGEs (advanced glycation end-products), which accumulate and form crosslinks among long-turnover proteins that are difficult to degrade [5]. It has also been reported that the formation of AGEs contributes to the “metabolic memory” phenomenon [6].

Overaccumulation of AGEs can lead to multiple diabetic complications in the body, such as damage to blood vessels in the eye resulting in diabetic retinopathy, peripheral nerve damage and impaired blood flow leading to diabetic neuropathy, and the development of various cardiovascular issues [7-9]. DN is also one of the major microvascular complications, primarily affecting kidney structures and the glomerular filtration unit of the body [7]. Foods cooked at high temperatures, processed foods rich in fats and sugar, smoking, and sedentary lifestyle habits promote increased production of AGEs in the body [10, 11].

Reactive precursors of AGE molecules interact with protein-bound Lys (lysine) or Arg (arginine) residues to form AGEs [12]. AGEs exert their effects primarily through a receptor-mediated pathway by binding to their receptor, RAGE (Receptor for Advanced Glycation End Products). RAGE consists of extracellular domains-namely the V domain, C1, and C2 domains-a transmembrane helix, and a short intracellular cytoplasmic tail (42 amino acids) [13]. AGEs interact only with the V domain of RAGE [9]. This interaction is mainly receptor-dependent and contributes to increased oxidative stress, activation of cellular protein factors, promotion of inflammation, cytokine signaling, cell adhesion, and tissue damage [14]. Therefore, managing AGEs could offer therapeutic potential beyond glycaemic control [7].

Screening metabolites from natural sources is one of the promising approaches in drug development. Plant-derived metabolites offer a wide range of bioactive properties, and their synergistic potential has shown beneficial effects in treating diseases with fewer adverse effects than synthetic drugs. *Curcuma caesia* (CC), a member of the Zingiberaceae family, is primarily reported for its antidiabetic properties [15]. Studies have reported the isolation of metabolites from the acetone and methanolic extracts of CC rhizomes [16]. Administration of the methanolic extract of CC rhizomes lowered fasting blood glucose and glycosylated haemoglobin levels in streptozotocin-induced Type 1 diabetic rats [17]. These findings encouraged us to investigate this potential herb with diverse bioactive properties. The novelty of this work lies in studying metabolites from CC rhizomes and evaluating their ability to mimic AGEs and disrupt AGE-RAGE interaction as a therapeutic approach in the progression and development of DN.

## 2 MATERIALS AND METHODS

### 2.1 Collection of Plant and Preparation of Plant Extract

The fresh rhizomes of *Curcuma caesia* (CC) were collected from Silchar, Assam (25.8465° N, 93.4299° E). The plant sample was authenticated by AUSCH (Assam University Silchar Central Herbarium), Silchar (Specimen Accession Number: 8563). The rhizomes were cleaned, made soil-free, shade-dried at room temperature, and then finely powdered before being subjected to the extraction procedure.

The extracts were prepared following a sequential extraction method using solvents of increasing polarity, namely Petroleum Ether (PE), Ethyl Acetate (EA), Acetone (Ac), and Methanol (ME) [18, 19]. A total of 100 g of dried powder was soaked in PE solvent for 72 hours and then filtered. The residue was dried and soaked in the next solvent, EA. This process was repeated for the subsequent solvents. The filtrates obtained from each solvent were dried and made solvent-free using a lyophilizer.

The four extracts prepared from *Curcuma caesia* rhizomes were designated as PE-CC (PE solvent), EA-CC (EA solvent), Ac-CC (Acetone solvent), and ME-CC (Methanol solvent).

### 2.2 Quantitative Phytochemical Analysis

#### 2.2.1 Total Phenolic Content

The total phenolic content (TPC) of *Curcuma caesia* was determined using the Folin-Ciocalteu (FC) technique with minor modifications [20, 21]. Initially, a 1 mg/mL plant extract was prepared in methanol. From this solution, 1 mL was taken and mixed with 100 µL of 10% FC reagent. After 3 minutes, 2 mL of 7.5% sodium carbonate was added, and the mixture was kept in the dark for 60 minutes. For TPC estimation, gallic acid was used as the standard. The optical density (OD) of the mixture was measured at 765 nm, and the results were expressed as gallic acid equivalents (GAE) in mg/g.

#### 2.2.2 Total Flavonoid Content

The total flavonoid content (TFC) of *Curcuma caesia* was estimated using the aluminum chloride method with slight modifications [22, 23]. In a test tube, 10 mg of plant extract was dissolved in 10 mL of methanol to prepare the stock solution. To this, 30 µL of 10% aluminum chloride solution, 30 µL of 5% sodium nitrate, and 200 µL of 1 mM NaOH solution were added sequentially. Subsequently, 640 µL of distilled water was added and mixed thoroughly. Then, 100 µL of the plant stock solution was added to the mixture and incubated in the dark for 15 minutes. Quercetin was used as the standard. Absorbance was measured at 415 nm, and the final TFC was expressed as quercetin equivalents (QE) in mg/g of the plant extracts.

#### 2.2.3 Antioxidant Activity

##### 2.2.3.1 DPPH (2,2-Diphenyl-1-picrylhydrazyl) Free Radical Scavenging Assay

The antioxidant activity of *Curcuma caesia* was evaluated using the DPPH free radical scavenging assay with slight modifications [24]. Initially, five different concentrations of each extract were prepared using the serial dilution technique. To prepare the DPPH solution, 4 mg of DPPH was dissolved in 50 mL of methanol to obtain a final concentration of 80 µg/mL.

For the assay, 200 µL of the DPPH solution was added to the diluted plant extract, and the volume was adjusted to 1 mL. The mixture was incubated in the dark for 30 minutes. Absorbance was then recorded at 517 nm using a spectrophotometer. Ascorbic acid was used as the reference standard, and IC_50_ values were determined from the concentration-response curve. The formula used for calculating the percentage inhibition is given by:

% DPPH radical scavenging activity= [(Ac-As)/Ac] *100

where Ac denotes the absorbance of the control, and As denotes the absorbance of the sample.

##### 2.2.3.2 ABTS (2,2’-azino-bis-(3-ethylbenzothiazoline-6-sulfonic Acid) Radical Scavenging Assay

The free radical scavenging activity of *Curcuma caesia* rhizome extracts was assessed using the ABTS free radical cation decolorization assay with minor modifications [25]. A 7 mM ABTS stock solution was prepared by dissolving 0.36 g of ABTS salt in 100 mL of distilled water. Separately, a 2.45 mM potassium persulfate solution was prepared by dissolving 0.066 g of potassium persulfate in 100 mL of distilled water. To generate the ABTS•^+^ radical cation, equal volumes of the 7 mM ABTS stock solution and the 2.45 mM potassium persulfate solution were mixed, and the mixture was incubated in the dark at 37 °C for 12-16 hours.

Prior to analysis, the ABTS•^+^ solution was diluted with methanol to obtain an absorbance of 0.7 at 734 nm. Ascorbic acid served as the reference standard. For the assay, 5 µL of sample extract was added to 200 µL of the diluted ABTS•^+^ solution and incubated in the dark for 10 minutes. Absorbance was measured at 734 nm, and the percentage inhibition was calculated using the formula:

% ABTS radical scavenging activity = (AB-As)/AB*100

where AB denotes the absorbance of the ABTS solution and As denotes the absorbance of the sample.

### 2.4 Metabolite Profiling of *Curcuma caesia*

Metabolite profiling is essential for identifying phytocompounds present in plant tissues using various analytical tools [26]. Liquid Chromatography-Mass Spectrometry (LC-MS) is one such technique widely employed for characterizing the phytochemical constituents of *Curcuma caesia* (CC) rhizome extracts.

The ME-CC extract sample was sent to the Sophisticated Instrumentation Facility (SIF), IIT Bombay, for metabolite analysis. Spectrometric analysis was performed using the Agilent Hunter software system. The methanolic fraction was analyzed using an Agilent LC system equipped with a column module, a hip sampler, and Agilent Jet Stream (AJS) technology. Chromatographic separation was achieved using a Q-TOF system with a dual ion source and a binary pump.

A 5 µL aliquot of the sample was injected, and the column temperature was maintained at 40 °C. Two solvent systems were used in the mobile phase: 0.1% formic acid in water (solvent A) and acetonitrile (solvent B). The flow rate was set to 0.300 mL/min. The optimized gradient elution profile is presented in Table **1**.

### 2.5 *In Silico* Pharmacokinetics Evaluation of the Metabolites

#### 2.5.1 Protein Receptor and Ligand Preparation

The crystal structure of the human RAGE protein was retrieved from the RCSB PDB database (https://www.rcsb.org). The PDB ID of the 3D structure obtained was **3O3U**, with a resolution of **1.50 Å**, and the structure was determined using the **X-ray diffraction** method. Energy minimization of the receptor protein was performed using **Swiss-PdbViewer version 4.1**, a free and user-friendly tool for visualizing and analyzing protein structures [27], available at https://spdbv.unil.ch/.

The SMILES format of all ligands was retrieved from the PubChem database (https://pubchem.ncbi.nlm.nih.gov/). The 3D conformers of the ligands were also downloaded from PubChem in **.sdf** format. Energy minimization of all ligands was conducted using **Avogadro**, a user-friendly molecular editing and visualization tool [28], available at https://avogadro.cc/.

Energy minimization of both the protein and ligands is essential to reduce potential energy and achieve the most stable conformations [29]. This step is crucial prior to molecular docking, as it ensures more accurate binding interactions between the ligands and the target molecule.

#### 2.5.2 Molecular Docking

The molecular interactions between the ligands and the protein target were studied using **Molegro Virtual Docker (MVD) version 6.0** software [30, 31]. The PDB file of the target protein was first loaded into the software interface, and all co-factors, water molecules, and bound ligands were removed. The 3D **.sdf** files of the ligands and positive controls were then imported.

The active site was identified using the *Detect Cavities* tool, and the docking site of the receptor was analyzed. For binding analysis, the grid box dimensions were set to **x: 33.31, y: 23.23, z: 75.03** with a **radius of 29**, and the grid resolution was set to **0.3 Å**. The search algorithm for energy minimization and hydrogen bond optimization was selected, and docking calculations were performed with **10 default runs for each compound** [32].

Post-docking analysis provided the docking scores for all ligand-protein interactions. Higher negative docking scores indicate stronger binding affinity of the ligands with the target protein [33].

#### 2.5.3 Prediction of Drug-likeness and ADME/Tox Properties

Lipinski's Rule of Five was predicted using the Molsoft L.L.C. webserver (https://molsoft.com/mprop/), a freely available online tool used for structural prediction, rational drug design, cheminformatics, and bioinformatics analysis [34]. ADME (Absorption, Digestion, Metabolism, Excretion) properties were predicted using the SwissADME webserver tool (http://www.swissadme.ch), which is used for computing physicochemical descriptors, pharmacokinetic features, and drug-likeness properties of molecules [35].

Toxicity prediction was carried out using the Protox 3.0 webserver, a free online tool accessible at https://tox.charite.de/protox3/. The prediction strategy is categorized into different toxicity levels, including organ toxicity, oral toxicity, toxicity targets, toxicological endpoints, and pathways [36]. SMILES notations were required for predicting the pharmacokinetics, physicochemical properties, drug-likeness, and toxicity profiles of the molecules.

#### 2.5.4 QSAR (Quantitative Structure-Activity Relationship)

Biologically active compounds were downloaded from ChEMBL (https://www.ebi.ac.uk/chembl). The data collected from ChemSketch software (ACD/ChemSketch, version 2021.1.2) and Molsoft (https://www.molsoft.com/mprop/) were used for descriptor generation. After preparing the compounds and descriptor list, EASY QSAR software was downloaded. It is a free web-based tool accessible at https://easyqsar.blogspot.com/, and it was used to predict the activity of the identified natural compounds. The activity was evaluated using the software-generated equation, and the IC_50_ values for the compounds were predicted.

## 3 RESULT

### 3.1 Methanolic Extract of CC has more Total Phenolic and Flavonoid Content and the Highest Antioxidant Activity

For 100 g of dried powder, the yield obtained was 5.98 g for PE-CC, 3.194 g for EA-CC, 0.404 g for Ac-CC, and 1.064 g for Me-CC, which were stored for further analysis. We measured TPC and TFC in all four solvent extracts and found that the ME-CC extract contained higher TPC and TFC than the other extracts. TPC was measured using the regression equation *y = 0.0001x + 0.013; R^2^ = 0.9935*, obtained from the gallic acid standard curve. The TPC value was 108.71 ± 5.0 mg GAE/100 g dry weight, and the TFC of ME-CC was found to be 248.49 ± 1.69 mg QE/100 g dry weight of the rhizomes, calculated from the regression equation *y = 0.0009x + 0.1693; R^2^ = 0.9807*, obtained from the quercetin standard curve.

We then measured the antioxidant activity of all extracts using DPPH and ABTS assays, as described in the methods section. Interestingly, the methanolic extract showed the highest antioxidant activity among the four extracts. The IC_50_ of ME-CC (percentage inhibition at 50%) for the DPPH assay was estimated to be 136.433 ± 3.60 µg/mL, and for the ABTS assay, it was found to be 89.36 µg/mL. The results of quantitative and antioxidant analyses of the four different extracts are provided in Table **S1**.

### 3.2 Metabolite Profiling of the Methanolic Extract of *Curcuma caesia* Rhizome

Considering the findings of TPC, TFC, and antioxidant activity, ME-CC was selected for phytochemical profiling using LC-MS. Phytochemical analysis revealed the presence of distinct and abundant secondary metabolites in the CC rhizomes. A total of 68 compounds were identified in the LCMS chromatogram of ME-CC. The chromatogram peaks of the identified metabolites from CC rhizomes are presented in Fig. (**1A** and **B**), and the mass spectra of the identified compounds with their mass-to-charge ratios and retention times (RT) are shown in Fig. (**2A** and **B**). The list of identified phytochemicals is provided in Table **S2**.

### 3.3 Binding Affinity with RAGE

In this study, we considered 32 AGE compounds, Vesperlysine A, Vesperlysine B, Vesperlysine C, GODIC, Pyrraline, MOLD, Argpyrimidine, CEA, CMA, CEL, N-carboxymethyllysine, DOGDIC, Methylglyoxal lysine dimer, MGH1, MGH2, Tetrahydropyrimidine, CMC, MGH3, GOLA, MODIC, Glucosepane, Formyline, Fructosyl-lysine, GDH1, GDH2, GH1, GH2, GH3, Carboxyethyl Cysteine, 5-Hydroxymethylfurfural, and 3-deoxyglucose-derived hydroimidazolone 1 [13]. Additionally, three known RAGE inhibitors, Azeliragon [37], FPS-ZM1 [38], and Pioglitazone [39], were included as positive controls, along with 68 identified ligands from ME-CC for molecular docking with RAGE.

Post-docking analysis revealed the MolDock scores for all docked compounds. Among the inhibitors, Azeliragon showed the highest docking score, while MODIC demonstrated the strongest binding to RAGE among all AGE compounds. From the 68 identified ligands, N1, N5, N10-Tris-trans-p-coumaroylspermine, Tetranactin, Lappaol-A, Piperaduncin-B, Marshdimerin, Arbekacin, and Pateamine exhibited the best binding efficiency with RAGE at low energy.

The MolDock scores of the best-docked ligands, positive controls, and RAGE inhibitors are presented in Table **2**. The chemical structures of the selected compounds are shown in Fig. (**3A**), and their binding poses and interactions with the target are displayed in Fig. (**3B**).

### 3.4 ADMET Analysis Revealed that Lappaol A and Piperaduncin B Exhibit Drug-like Properties

After molecular docking analysis, seven compounds were selected for ADME evaluation using the SwissADME webserver. ADME profiling revealed that **Lappaol A** and **Piperaduncin B** showed a higher probability of intestinal absorption compared to the remaining five compounds. Log P (the logarithm of the partition coefficient of a substance in octanol versus water) measures the lipophilicity of a compound and is an important factor for oral drug absorption [40]. The optimal Log P value typically ranges between 0 and 5 (Table **3**) [41].

Using Protox 3.0, the Lethal Dose 50 (LD50) values of the compounds were predicted in mg/kg and categorized into toxicity classes:

Class 1 (LD50 ≤ 5 mg/kg; fatal if swallowed),

Class 2 (5 ≤ LD50 ≤ 50 mg/kg; fatal if swallowed),

Class 3 (50 ≤ LD50 ≤ 300 mg/kg; toxic if swallowed),

Class 4 (300 ≤ LD50 ≤ 2000 mg/kg; harmful if swallowed),

Class 5 (2000 ≤ LD50 ≤ 5000 mg/kg; may be harmful if swallowed),

Class 6 (LD50 > 5000 mg/kg; non-toxic) [42].

The results are presented in Table **4**.

Out of the seven compounds, six were predicted to be nephrotoxic, while Marshdimerin was predicted to be non-toxic with respect to nephrotoxicity. Although the nephrotoxicity probability scores were relatively low for most compounds (50-65%), Arbekacin showed a higher probability score (91%). Both Lappaol A and Piperaduncin B exhibited higher LD50 values, and their prediction accuracy (68.07%) suggests a relatively high likelihood of correctly predicting the toxicity range.

In summary, the ADME-Tox analysis indicates that Lappaol A and Piperaduncin B possess favorable pharmacological characteristics and may serve as potential drug candidates for treating DN.

### 3.5 QSAR (Quantitative Structure-Activity Relationship) Analysis for Lappaol A and Piperaduncin B

QSAR predicts the properties of a molecule using mathematical models that correlate activity with its molecular features. Based on the selected nine descriptors and the analysis of 21 training data points, the model showed an R^2^ value of 96.77%, indicating a strong fit between the equation and the given data. Furthermore, the small difference between R^2^ (96.77%) and adjusted R^2^ (94.12%) suggests a robust overall prediction. The predicted IC_50_ values of the test compounds are shown in Table **5**.

## 4 DISCUSSION

Polyphenols can effectively reduce glycation through multiple mechanisms such as decreasing oxidative stress, inhibiting the formation of Amadori products and dicarbonyl groups, and modulating cellular signaling pathways [43]. One of the key properties of polyphenols is their antioxidant ability to neutralize reactive oxygen species [44]. *Curcuma caesia* is a polyphenol-rich herb with notable pharmacological activities [45], and the traditional uses of the *Curcuma* genus in treating various ailments have been studied for many years [46]. Reported studies on the methanolic extract of *Curcuma caesia* (ME-CC) rhizomes have demonstrated the presence of phenols and flavonoids [47-49].

The antioxidant activities of PE, EA, Ac, and ME extracts of *Curcuma caesia* show their capability to scavenge free radicals in in-vitro DPPH and ABTS assays, with the highest activity observed in the ME-CC extract. Similar quantitative assessments of flavonoids, phenols, and antioxidant activity of *Curcuma caesia* polar extracts have also been reported, showing very low IC_50_ values [50, 51].

Lappaol A, along with six other compounds isolated from the seeds of *Arctium lappa*, exhibited prominent antioxidant and anti-aging activities, upregulated *jnk-1* expression, and promoted longevity and stress resilience in *Caenorhabditis elegans* [52]. Piperaduncin B has been isolated from the leaves of *Piper aduncum* [53], and antioxidant activity has been reported in ethanolic extracts of *Piper aduncum* leaves [54].

Activation of signaling pathways occurs when exogenous and endogenous AGEs bind to RAGE, influencing the progression of multiple diseases [55]. In reported studies, binding of RAGE with its ligands has been shown to interfere with the development of diabetic complications [6]. Drugs that inhibit AGEs formation have been associated with certain side effects [56]. Therefore, it is essential to search for biologically active natural compounds that can bind efficiently with the RAGE target while exhibiting minimal side effects.

Molecular docking analysis of ligands, using AGEs as positive controls, identified molecules with better binding scores than both the positive controls and the known inhibitors. Lappaol A showed a docking score of -179.297 with RAGE, substantially higher than both MODIC (the positive control) and Azeliragon (a known inhibitor). We also observed that Piperaduncin B exhibited a higher docking score than MODIC and Azeliragon. Thus, we predict that both Lappaol A and Piperaduncin B could act as AGEs-mimicking natural compounds.

A similar study reported that compound 62 showed high binding affinity with the RAGE protein and was proposed as an inhibitor of diabetic retinopathy and related vascular complications [57]. Another study suggested that a herbal formulation from *Schisandra chinensis* fruit exhibited stable binding affinity to the RAGE protein and could regulate the AGE-RAGE signaling mechanism [58].

Overall, molecular docking analysis confirmed that the identified metabolites have strong binding affinity for the target protein and can potentially block the receptor from binding AGEs molecules, thereby preventing downstream signaling events and the progression of diabetic nephropathy (DN).

Pharmacokinetic studies using ADMET parameters further predicted good absorption of these compounds in the digestive tract upon oral consumption. In our study, the compounds showed no blood-brain barrier permeability and adhered to RO5 (Rule of Five) drug-likeness criteria with minimal violations, as shown in Table **3**. Lipinski and his coworkers at Pfizer formulated these rules to evaluate whether a molecule exhibits “drug-like” properties [59]. These rules were originally developed to guide the design of synthetic compounds with favorable biophysical properties and oral bioavailability [60].

According to the RO5, a bioactive molecule must fulfill specific criteria to be considered suitable as an orally administered drug. For good intestinal absorption, a molecule should have a molecular mass < 500 Da, hydrogen bond donors (HBD) ≤ 5, hydrogen bond acceptors (HBA) ≤ 10, and a Log P value < 5 [61]. However, Lipinski clearly excluded these rules for natural compounds, as many bioactive natural products can maintain low hydrophobicity and high molecular mass with numerous rotatable bonds [62], and they often enter cells through membrane transporters rather than passive diffusion [60].

The Log P values of the compounds obtained ranged from -5.4 to 5. Higher Log P values indicate greater lipid affinity, reflecting the lipophilic nature of a compound [63]. Lipophilic compounds can penetrate the lipid bilayer of cellular membranes and facilitate absorption in the gastrointestinal tract [64]. Therefore, lipophilicity is essential for optimal oral absorption of a drug candidate.

Toxicity assessment of Lappaol A and Piperaduncin B indicated a moderate range of nephrotoxicity, with LD50 values between 300 and 2000 mg/kg, categorized as class 4, meaning they are considered harmful if swallowed. Overall, ADMET analysis confirmed good intestinal absorption, favorable pharmacokinetic properties, and moderate toxicity for both Lappaol A and Piperaduncin B, supporting their potential as lead compounds for therapeutic use.

QSAR analysis showed an Rsq value of 96.77% for RAGE (Fig. **4**) and predicted favorable IC_50_ values for the identified compounds (Table **5**). Previous QSAR studies on polyphenols have effectively predicted and evaluated their potential as antioxidative compounds [65]. Researchers have also applied QSAR models to study compounds targeting GPR40, α-glucosidase inhibitors, flavone derivatives [66], coumarin derivatives [67], and andrographolide derivatives [68], providing insights into their therapeutic potential for treating diabetes [69].

The rationale of this study was to identify therapeutic compounds capable of mimicking AGEs and controlling the progression of DN by inhibiting RAGE. Virtual screening of the identified compounds through molecular docking and ADMET analysis revealed that Lappaol A and Piperaduncin B bind effectively with RAGE and may interrupt the signaling cascades normally triggered by RAGE activation, a characteristic of toll-like receptors. Therefore, Lappaol A and Piperaduncin B, with their strong drug-likeness and favorable ADMET characteristics, are proposed for further *in vivo* studies and eventual clinical trials toward developing new therapeutic agents for regulating the progression and treatment of diabetic complications.

## CONCLUSION

The present study identified Lappaol-A and Piperaduncin-B from the methanolic extract of *Curcuma caesia* as AGE-mimicking natural compounds capable of inhibiting the binding of AGEs to their receptor, RAGE. This inhibition may help prevent the activation of signaling pathways associated with tissue damage and the progression of diabetic nephropathy. Computational analyses, including molecular docking, ADMET profiling, and QSAR studies, indicated that these compounds comply with drug-likeness criteria and exhibit favorable pharmacokinetic properties. Their drug-like characteristics suggest strong potential for further exploration in pharmaceutical research. However, additional validation through *in vitro* and *in vivo* experiments is required to establish their therapeutic efficacy. Future studies should focus on dose optimization and other pharmacological parameters to determine their suitability as therapeutic agents for diabetic nephropathy and its related complications.

## AUTHORS' CONTRIBUTIONS

The authors confirm their contributions to the paper as follows: P.N.: Contributed to data curation, data analysis, and writing of the original draft and its preparation; A.K.P.: Contributed to writing the original draft, as well as reviewing and editing the manuscript; R.N., M.D., R.N. and D.N.: Contributed to reviewing and editing the manuscript; J.S.: Contributed to the investigation and to reviewing and editing the manuscript; S.K.: Contributed to writing the original draft, performing formal analysis, and reviewing and editing the manuscript. ADT contributed to the conceptualization, visualization, methodology, investigation, supervision, software, and to reviewing and final editing of the manuscript. All authors reviewed the results and approved the final version of the manuscript.

## Figures and Tables

**Fig. (1):**
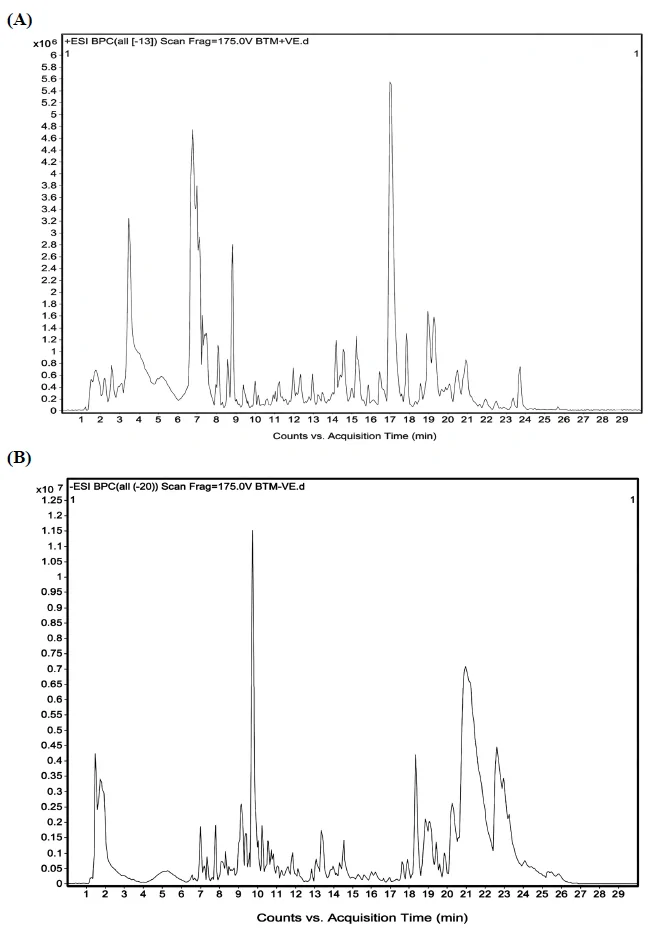
Representation of LC-MS chromatogram peaks for the compounds identified from methanolic extract-based ESI positive in (**A**) and methanolic extract-based ESI negative analysis in (**B**).

**Fig. (2):**
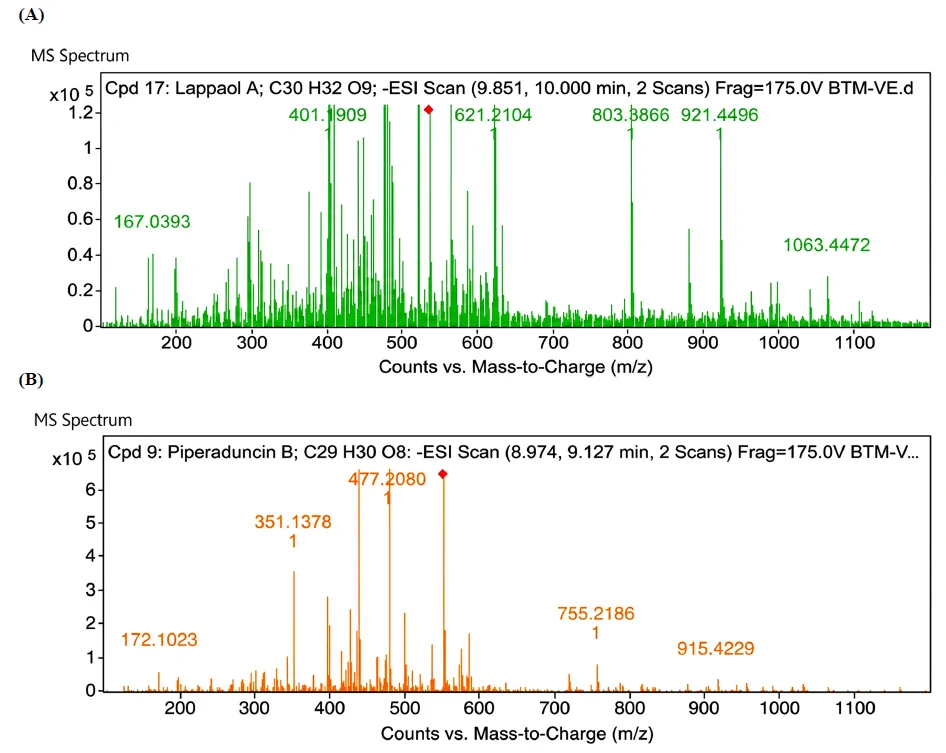
**(A)** Mass spectrum of compounds Lappaol A with mass-charge ratio of 531.194 at RT to be 9.94. **(B)** Mass spectrum of compound Piperaduncin B with mass-charge ratio of 551.188 at RT to be 9.04. *The red dot in the images shows the presence of these identified metabolites in the ME-*CC* rhizome sample.

**Fig. (3):**
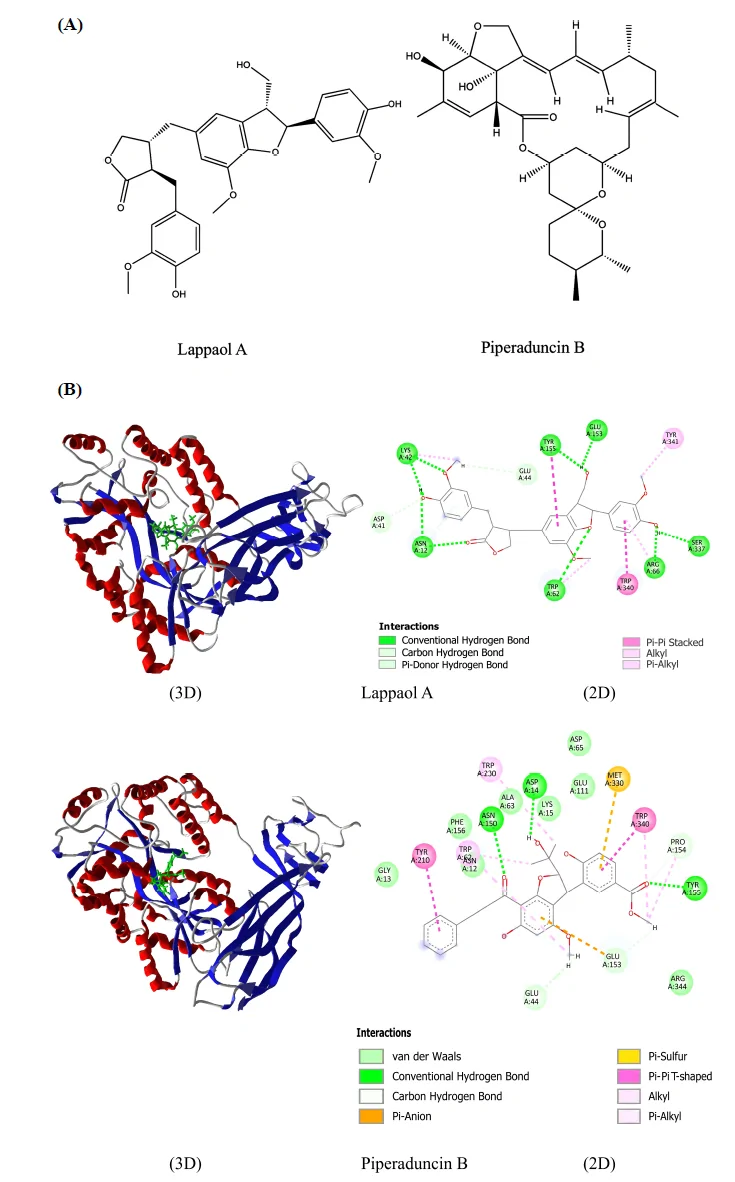
Chemical structure, 3D interaction - 2D poses of Lappaol-A, Piperaduncin B docked with the target protein RAGE. Different bond interactions with amino acid residues are visible in 2D images of the compounds.

**Fig. (4):**
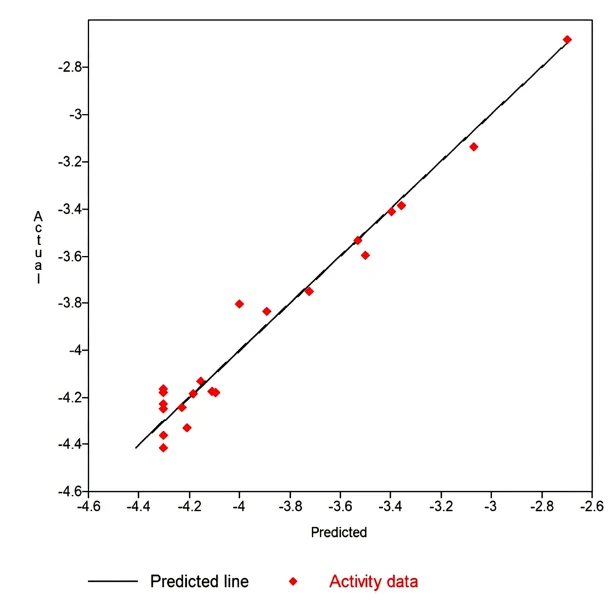
QSAR plot.

**Table 1:** Time and solvent gradient used for LC-MS analysis.

**Sl No.**	**Time (min)**	**Solvent A** **(Water)**	**Solvent B** **(Acetonitrile)**	**Rate of Flow** **mL/min**	**Pressure (bar)**
1	1.00	95%	5%	0.300	1200
2	25.00	0%	100%	0.300	1200
3	30.00	0%	100%	0.300	1200
4	31.00	95%	5%	0.300	1200
5	35.00	95%	5%	0.300	1200

**Table 2:** MolDock and H-bond score of best docked compounds.

**Target**	**Ligands**			**Positive Control**			
	**Name**	**MolDock Score**	**H-bond**	**Name**	**MolDock Score**		**H-bond**
RAGE	N1, N5, N10-Tris-trans-p-coumaroylspermine	-188.443	-4.121	AGEs			
	Lappaol A	-177.377	-5.092	MODIC	-137.710		-7.578
	Marshdimerin	-175.443	-1.209				
	Arbekacin	-168.888	-12.403				
	Tetranactin	-168.704	-1.049	**RAGE Inhibitor**			
	Pateamine	-165.581	-1.034	Name		MolDock Score	H-bond
				Azeliragon		-140.980	0
	Piperaduncin B	-161.581	-5.064				

**Table 3:** Pharmacokinetic properties of the compounds from the SwissADME webserver.

**Parameters**	**Names of the Ligands**						
	**N1, N5, N10-Tris-trans-p-coumaroylspermine**	**Lappaol A**	**Marshdimerin**	**Arbekacin**	**Tetranactin**	**Pateamine**	**Piperaduncin B**
Molecular weight	640.77	536.57	758.94	552.62	793.04	555.77	506.54
Hydrogen bond acceptor	7	9	8	15	12	7	8
Hydrogen bond donor	6	3	0	11	0	1	3
Mol Log P	3.62	2.8	13	-5.4	8.35	2.45	5
Lipinski #violations	1	1	2	3	2	0	1
GI absorption	Low	High	Low	Low	Low	High	High
TPSA	156.43	123.91	97.34	297.27	142.12	122.99	122.52
BBB permeant	No	No	No	No	No	No	No
P-gp substrate	No	Yes	Yes	Yes	Yes	Yes	Yes

**Table 4:** Lethal Dose 50 and Organ toxicity (Nephrotoxicity) prediction using Protox 3.0.

**Sl. No.**	**Ligand Names**	**Molecular Weight**	**Nephrotoxicity** **level**	**Probability (%)**	**Predicted LD50 (mg/kg)**	**Predicted Toxicity Class**	**Prediction Accuracy**
1	N1,N5,N10-Tris-trans-p-coumaroylspermine	640.77	Moderately toxic	Active (54)	500	4	69.26
2	Lappaol A	536.57	Moderately toxic	Active (65)	1500	4	68.07
3	Marshdimerin	758.94	Slightly toxic	Inactive (58)	2500	5	67.38
4	Arbekacin	552.62	Slightly toxic	Active (91)	5000	5	100
5	Tetranactin	793.04	Slightly toxic	Active (57)	2500	5	100
6	Pateamine	555.77	Toxic	Active (50)	200	3	3
7	Piperaduncin B	506.54	Moderately Toxic	Active (54)	2000	4	68.07

**Table 5:** Adjusted Rsq, Rsq value, and Equation of the QSAR plot.

	**Rsq**	Adjusted Rsq*	**F** **Statistics**	**Critical F**
**RAGE**	96.77%	94.12%	36.56	2.39
**Equation**				**Predicted IC_50_** (Test data)
**RAGE**	**Activity** =(-5.51E+001)-5.23E-002*(FormulaWeight)-1.27E-001*(MolarRefractivity)-4.11E-002*(MolarVolume)+ 5.47E-002*(Parachor)+1.93E+001*(IndexofRefraction)-3.31E-001*(SurfaceTension)+3.10E+001*(Density)+ 6.09E+022*(Polarizability)-1.68E-001*(RDBE)			Lappaol A =467.73nM (0.46µM) Piperaduncin B =933.25nM (0.93 µM)

*Rsq value should be high for a good QSAR equation. Higher Rsq indicates better fitting of the equation to the given data, suggesting it will provide better predictions for new test data. F-statistics should be greater than the Critical F value. A smaller difference between Rsq and Adjusted Rsq indicates a stronger overall prediction.

## Data Availability

The data and supportive information are available within the article.

## LIST OF ABBREVIATIONS

ABTS: 2,2’-Azino-bis-(3-ethylbenzothiazoline-6-sulfonic acid)
ADME/Tox: Absorption, Digestion, Metabolism, Excretion, Toxicity
AGEs: Advanced Glycation End Products
Ace-CC: Acetone extract of Curcuma caesia
*C. caesia*: *Curcuma caesia*
COX-1: Cyclooxygenase-1
DALYs: Disability-Adjusted Life Years
DN: Diabetic Nephropathy
DPPH: 2,2-Diphenyl-1-picrylhydrazyl
EA-CC: Ethyl acetate extract of Curcuma caesia
ESRD: End-Stage Renal Disease
GHS: Globally Harmonized System
g: Gram
LCMS: Liquid Chromatography-Mass Spectrometry
ME-CC: Methanol extract of Curcuma caesia
MVD: Molegro Virtual Docker
NCBI: National Center for Biotechnology Information
PE-CC: Petroleum ether extract of Curcuma caesia
QSAR: Quantitative Structure-Activity Relationship
RAGE: Receptor for Advanced Glycation End Products
SAIF: Sophisticated Instrumentation Facility
TFC: Total Flavonoid Content
TPC: Total Phenolic Content
