# Supplementary material for: Identification of RAGE Inhibitors from Curcuma caesia Roxb: Implications in Treatment against Diabetic Complications
Source: Curr Diabetes Rev. 2026 Jan 20;22(8):E15733998416568. doi: 10.2174/0115733998416568251122070701 (PMC13717578; doi:10.2174/0115733998416568251122070701)
Supplement: Supplementary file 1 [file CDR-22-8-E15733998416568_SD1.pdf]

Supplementary Material

Identification of RAGE Inhibitors from Curcuma caesia Roxb: Implications in Treatment against Diabetic Complications

Priyakshi Nath[1], Anil K. Pasupulati[2], Rupshikha Nath[1], Rajat Nath[3], Mriganka Das[1], Deepa Nath[4], Jagajjit Sahu[5], Sibashish Kityania[1,*] and Anupam Das Talukdar [1,*]

[1] Department of Life Science and Bioinformatics, Assam University, Silchar, Cachar, Assam, 788011, India; [2]Department of Biochemistry, University of Hyderabad, 500046, India; [3]Department of Biotechnology and Microbiology, School of Natural Sciences, Techno India University Tripura (TIUT), Agartala, 799004, Tripura; [4]Department of Botany, Gurucharan College, Silchar, Cachar, Assam, 788004, India; [5]Bioinformatics Nexus Network (BNSquare), GyanArras Academy, Bhubaneshwar, Khurda, Odisha, 751003, India

Table S1. Values of Antioxidant and quantitative assay of *Curcuma caesia* Roxb. using four different solvents.

Assays ⇨ / ⇩ Extracts	TPC (mg GAE/100g)	TFC (mg QE/100g)	DPPH Assay (µg/ml)	ABTS Assay (µg/ml)
**PE-CC**	76.03±3.8	106.6±2.2	202.5±2.6	303.57
**EA-CC**	70.6±2.1	127.7±2.9	196.4±3.4	214.62
**Ac-CC**	72.26±4.4	133.3±1.9	186±4.4	191.66
**Me-CC**	108.71± 5.0	248.49 ± 1.69	136.433 ± 3.60	89.36

Table S2. List of metabolites identified from LC-MS analysis of *Curcuma caesia* Roxb.

Name of the Plant	Plant parts used	Process of Screening used	Metabolites Identified	Mass	Retention Time	m/z
Curcuma caesia	Rhizome	HR-LCMS/MS(Q-TOF)	1-(Indol-3-yl) propanol 3-phosphate	255.0702	2.252	256.0776
			2-Methylpiperidine	99.1046	3.551	122.0938
			gamma-Coniceine	125.1198	6.856	148.109

			p-Aminobenzamidine	135.0771	6.868	136.0843
			(S)-2,3,4,5-Tetrahydropiperidine-2-carboxylate	127.0628	6.896	150.052
			Phenylboronic acid	121.0568	6.928	144.0453
			Coniine	127.1356	7.43	150.1248
			N(alpha)-t-Butoxycarbonyl-L-leucine	231.1452	7.438	254.1345
			Methyl N-methylanthranilate	165.0785	8.137	188.0675
			2-Ethyl-5-methylpyridine	121.0891	8.846	144.0784
			Riesling acetal	226.1558	9.062	249.1452
			2,4-Bis(acetamido)-2,4,6-trideoxy-beta-L-altropyranose	246.1216	9.24	247.1298
			Ketosantalic acid	250.1563	9.555	251.1644
			5-Methylcytidine	257.1009	9.773	258.1088
			3-[[5-Methyl-2-(1-methylethyl)cyclohexyl]oxy]-1,2-propanediol	230.1863	10.288	253.1756
			11-Hydroxy-9-tridecenoic acid	228.1699	10.422	251.1601
			hexamethylene bisacetamide	200.153	10.52	201.1603
			Serinyl-Threonine	206.0906	10.706	207.0978
			Dexmedetomidine	200.1347	11.26	201.142
			Ambronide	236.2119	11.635	259.2013
			Herculin	251.2228	11.884	274.2121
			3-Epinobilin	346.1759	11.967	369.165
			Bakkenolide C	348.1906	12.024	371.1799
			3-Methylbutyl 2-furanbutanoate	224.1401	12.233	247.1293
			(R*,S*)-4-[1-Ethyl-2-(4-fluorophenyl)butyl]phenol	272.1604	12.817	295.1496
			Suberylglycine	231.1093	13.775	254.0986
			(Z)-1,5-Tridecadiene	180.1869	14.239	203.1761

			1-Tridecene	182.2025	14.749	205.1918
			4-(Butoxymethyl)phenol	180.114	14.86	203.1033
			2-Deoxystreptidine	246.1396	14.936	247.1469
			gamma-L-Glutamyl-L-pipecolic acid	258.1216	15.246	259.1289
			Actinamine	206.1269	15.374	207.1341
			16-Oxo-palmitate	270.2167	16.086	293.2062
			16-Hydroxy hexadecanoic acid	272.2327	16.473	295.2218
			2-Methylundecanal	184.1815	16.567	207.1708
			Geijerone	178.1347	16.976	201.124
			Metharbital	198.1009	17.09	199.1079
			Slaframine	198.1375	18.676	199.1447
			Isoleucyl-Alanine	202.1321	18.681	203.1394
			(3Z,6Z,9Z)-dodecatrienol	180.1505	18.992	203.1398
			Caffeine	194.0802	4.236	253.094
			3-Dehydro-L-gulonate	194.0431	1.603	193.036
			Vat Yellow 4	332.0809	6.999	331.0736
			Gambirtannine	330.1378	7.096	329.1306
			Piperaduncin B	506.1907	8.691	551.1892
			Ailanthinone	478.2144	8.989	477.2079
			2-Methoxyestrone 3-glucuronide	476.2003	9.419	475.1931
			Armillyl orsellinate	402.1984	9.598	401.1911
			Marshdimerin	758.3885	9.779	803.3872
			Lappaol A	536.2019	9.945	535.1946
			Cinegalline	430.2064	9.966	475.2048
			Silandrin	466.1219	10.059	465.1147
			Isosyringinoside	534.187	10.066	533.1795
			Alvimopan	424.2311	11.659	483.2454

			O-Acetylcypholophine	360.2031	11.893	405.2017
			5-Acetylamino-6-formylamino-3-methyluracil	226.0703	13.165	271.0684
			N1, N5, N10-Tris-trans-p-coumaroylspermine	640.3179	13.549	685.3161
			Trichilin A	674.2806	14.593	719.2792
			Pateamine	555.3204	18.686	554.3146
			Kiwiionoside	406.2253	19.067	465.239
			Arbekacin	552.3159	19.286	551.3105
			Milbemectin	528.3138	20.24	527.3078
			Philanthotoxin 343	435.322	20.342	480.3202
			Formimidoyl-fortimicin A	432.2726	20.444	477.2728
			Fumigaclavine A	298.1677	21.169	297.1608
			Atenolol	266.1618	21.171	265.1548
			6,8a-Seco-6,8a-deoxy-5-oxoavermectin "1b" aglycone	554.3314	21.241	553.3263
			Phlegmarine	250.237	22.079	295.2352
			Aprindine	322.2353	22.425	321.228
			Tetranactin	792.5037	22.718	851.5181
			Hydroquinidine	326.1992	25.392	325.1923
